# Durable responses and reversible toxicity of high-dose interleukin-2 treatment of melanoma and renal cancer in a Community Hospital Biotherapy Program

**DOI:** 10.1186/2051-1426-2-13

**Published:** 2014-05-14

**Authors:** Roxanne Payne, Lyn Glenn, Helena Hoen, Beverley Richards, John W Smith, Robert Lufkin, Todd S Crocenzi, Walter J Urba, Brendan D Curti

**Affiliations:** 1 Providence Cancer Center, Providence Portland Medical Center, Earle A. Chiles Research Institute, 4805 NE Glisan Street, Portland, OR 97213, USA; 2 Compass Oncology, 265 N Broadway, Portland, OR 97725, USA

**Keywords:** Interleukin-2, Melanoma, Renal cancer

## Abstract

**Background:**

High-dose interleukin-2 (IL-2) has been FDA-approved for over 20 years, but it is offered only at a small number of centers with expertise in its administration. We analyzed the outcomes of patients receiving high-dose IL-2 in relation to the severity of toxicity to ascertain if response or survival were adversely affected.

**Methods:**

A retrospective analysis of the outcomes of 500 patients with metastatic renal cell carcinoma (RCC) (n = 186) or melanoma (n = 314) treated with high-dose IL-2 between 1997 and 2012 at Providence Cancer Center was performed. IL-2 was administered at a dose of 600,000 international units per kg by IV bolus every 8 hours for up to 14 doses. A second cycle was administered 16 days after the first and patients with tumor regression could receive additional cycles. Survival and anti-tumor response were analyzed by diagnosis, severity of toxicity, number of IL-2 cycles and subsequent therapy.

**Results:**

The objective response rate in melanoma was 28% (complete 12% and partial 16%), and in RCC was 24% (complete 7% and partial 17%). The 1-, 2- and 3-year survivals were 59%, 41% and 31%, for melanoma and 75%, 56% and 44%, for RCC, respectively. The proportion of patients with complete or partial response in both melanoma and RCC was higher in patients who a) required higher phenylephrine doses to treat hypotension (p < 0.003), b) developed acidosis (bicarbonate < 19 mmol (p < 0.01)), or c) thrombocytopenia (<50, 50–100, >100,000 platelets; p < 0.025). The proportion achieving a complete or partial response was greater in patients with melanoma who received 5 or more compared with 4 or fewer IL-2 cycles (p < 0.0001). The incidence of death from IL-2 was less than 1% and was not higher in patients who required phenylephrine.

**Conclusions:**

High-dose IL-2 can be administered safely; severe toxicity including hypotension is reversible and can be managed in a community hospital. The tumor response and survival reported here are superior to the published literature and support treating patients to their individualized maximum tolerated dose. IL-2 should remain part of the treatment paradigm in selected patients with melanoma and RCC.

## Background

Metastatic melanoma and metastatic RCC are both highly lethal tumors with a poor prognosis. The incidence and prevalence of both cancers are increasing in the United States. In 2013 there are estimated to be greater than 77,000 new diagnoses and 9500 deaths from melanoma and approximately 65,000 new diagnoses with 13,500 deaths from RCC. Only 5%-10% of patients survive for 5 years once metastatic disease develops [1].

There have been recent advances in the treatment of melanoma. Two randomized phase III studies, which showed improved survival for patients with advanced melanoma treated with ipilimumab led to the FDA approval of ipilimumab for first or second-line treatment of metastatic melanoma in March 2011 [2,3]. Long term follow-up data has also been reported after ipilimumab in 1861 patients revealing a 5 year survival of 22% [4]. Monotherapy with PD-1-directed antibodies and combinations of T-cell check point inhibitors are also showing significant clinical promise. Nivolumab (anti-PD-1) and ipilimumab showed an objective response rate of 40% in patients with metastatic melanoma [5]. Even with the recent FDA approval of ipilimumab showing a 4 month improvement in median survival, [2] and targeted agents such as vemurafenib having a high initial response rate of approximately 50%, [6,7] 90% of patients with widespread melanoma die within 5 years using extant therapy.

There has also been significant progress in the development of new agents for the treatment of metastatic renal cancer (RCC). Targeted agents approved for advanced RCC include sorafenib, sunitinib, pazopanib, temsirolimus, everolimus and axitinib [8-12]. Even though these agents have improved treatment of patients with metastatic kidney cancer, VEGF-TKI or m-TOR directed therapies are associated with a median duration of response of approximately 11 months. Median survival reported with VEGF-TKI therapy is generally less than 2 years, although a minority of patients can achieve control of disease for several years by using these agents in sequence. Currently available oral agents for RCC do not cure metastatic disease.

Interleukin-2 (IL-2) is a cytokine produced by activated T cells that increases proliferation and activation of cytotoxic T-cells, NK cells and monocytes [13]. The antitumor activity of recombinant IL-2 in preclinical and clinical settings) led to 7 pivotal clinical trials and FDA approval for patients with metastatic kidney cancer in 1992 and metastatic melanoma in 1998. Overall response was 16% in melanoma and 15% in RCC. Long-term survival was also demonstrated in a minority of patients with melanoma and RCC; however, no prospective randomized phase 3 studies have been performed with IL-2 showing a survival benefit. Despite the absence of phase 3 studies, IL-2 was approved because of durable responses were observed, and at the time of approval there were no other better therapeutic alternatives in melanoma and RCC. IL-2 toxicity depends on the dose, route and duration of administration. High-dose bolus IL-2 has systemic effects that can impact all organ systems profoundly. These effects are due to a vascular leak syndrome initiated by circulating cytokines, inducible nitric oxide, and activation of neutrophils, complement and the endothelium [14,15]. In particular, patients may experience profound hypotension, acute renal injury, acidosis and other metabolic disturbances. The use of high-dose bolus IL-2 remains limited because of its toxicity and relatively low response rates; however, the durable responses are clinically meaningful and IL-2 has a place in recently published treatment guidelines for both melanoma and renal cancer [16-18].

We report on the clinical outcomes of 500 patients with melanoma and RCC treated with high-dose IL-2 at our cancer center. The response and survival we observed is superior to historical data for IL-2 and our analysis supports that treating patients to their individualized maximum tolerated dose (MTD) enhances response. We also demonstrate that there is no adverse influence on survival or response by the severity of toxicity.

## Results

### Patient characteristics

The 1601 admissions in this retrospective analysis represent 500 consecutive patients treated at the Providence Cancer Center Biotherapy Program from 1997 to 2012 are summarized in Table 1. Seven other patients in our database were excluded due to missing response information or IL-2 offered in the adjuvant setting via a clinical trial.

**Table 1:** Summary of patient characteristics, including sites of metastatic disease

	**Melanoma (%) N=314**	**Renal cell carcinoma (%) N=186**
**Gender**		
Male	187 (60)	138 (74)
Female	127 (40)	48 (26)
**Age**		
Mean (SD)	53.2 (11.9)	57.3 (9.1)
Range	20 -79	30 – 80
**ECOG performance status**		
0	220 (71)	136 (73)
1	85 (27)	47 (25)
2	7 (2)	3 (2)
**Sites of metastatic disease**		
Lymph node	154 (49)	39 (21)
Lung	181 (58)	137 (74)
Liver	96 (31)	31 (17)
Bone	79 (25)	43 (23)
Brain	47 (15)	14 (7)
Subcutaneous	69 (22)	1(1)
**Number of metastatic sites**		
1	42 (13)	61 (33)
2	69 (22)	50 (27)
3	54 (17)	38 (20)
4	149 (48)	37 (20)
**Prior treatment**		
Nephrectomy	2 (1)	162 (87)
Radiation	139 (44)	41 (22)
Chemotherapy	28 (9)	8 (4)
Immunotherapy	119 (38)	9 (5)
VEGF TKI	0 (0)	11 (6)
Other	71 (23)	24 (13)

The majority of the patients with melanoma treated with prior immunotherapy received interferon in the adjuvant setting. Six patients with melanoma received ipilimumab and 3 received vemurafenib before IL-2. Of the patients with melanoma, two patients had choroidal and 14 mucosal primary sites. Twenty-four patients had an LDH >500. Two patients with biopsy proven metastatic melanoma also had a nephrectomy, one individual had a previous history of localized renal cancer treated surgically and the other had melanoma metastatic to the kidney in whom nephrectomy was performed for palliation. Patients with brain metastases could receive IL-2 if they were treated with surgery, radiation or the combination, and were asymptomatic and off steroids. The distribution of metastatic sites, age and gender were as expected based on the natural history of these malignancies. Functional status was normal (ECOG 0) for > 70% of patients who received high dose IL-2 on our Biotherapy Service.

### Clinical outcomes

**Figure 1:**
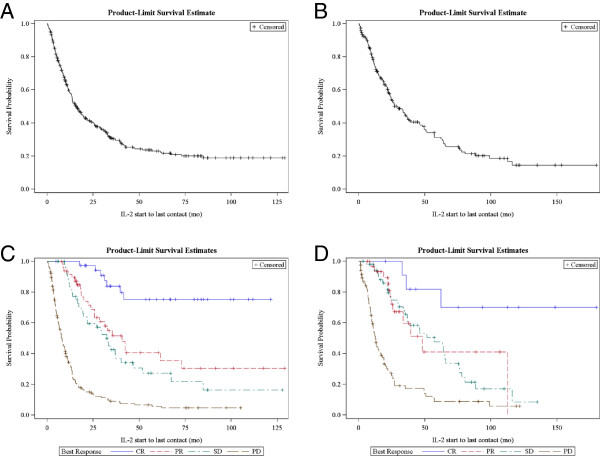
**Kaplan-Meier (product limit) survival for melanoma (A) and RCC (B). **Survival by best response as assessed by the treating physician in melanoma (C) and RCC (D).

Median follow-up was 4.7 years and ranged from 1 month to 10.8 years for patients with melanoma. For patients with RCC, median follow-up was 7.1 years and ranged from 1 month to 15 years at the time of the database analysis. The objective response rate in melanoma was 28% (complete 12% (n = 37) and partial 16% (n = 49), and in RCC was 24% (complete 7% (n = 12) and partial 17% (n = 32)). Stable disease was observed in 51 patients with melanoma and 54 with renal cancer. We observed melanoma regression in patients with poor clinical prognostic indicators. For instance, among the 24 patients who had an LDH > 500 IU there were 2 CR, 2 PR and 2 SD. Table 2 shows the percent overall survival for years 1 – 5 after treatment. Figure 1 shows survival by response group. The median survival of patients achieving a complete response (CR) was not reached in melanoma or RCC. For patients with partial response (PR), stable disease (SD) or progressive disease (PD), the median survivals were 40.7, 32.6 and 7.7 months in melanoma, and 48.1, 57.2 and 12.7 in RCC, respectively. The survival of patients with PR or SD and subsequent progression after IL-2 was influenced by other systemic therapies (see below).

**Table 2:** Percent survival by year and diagnosis after IL-2

**Year**	**1**	**2**	**3**	**4**	**5**
Melanoma	59	41	31	24	23
Renal cancer	75	56	44	39	31

**Figure 2:**
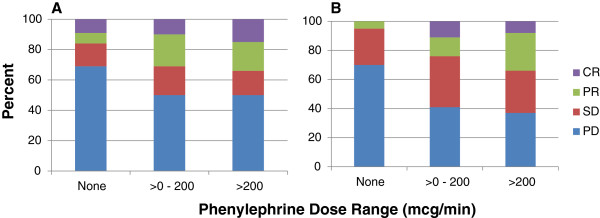
Percent response by phenylephrine dose range in melanoma (A) and RCC (B).

Responses were analyzed by the severity of toxicity. We chose to perform this analysis to ascertain if either response or survival was influenced by the main dose-limiting toxicity of IL-2, namely, hypotension, occurring during any treatment cycle. Phenylephrine is the pressor agent used routinely on our Biotherapy Service and pressor dose is titrated to maintain blood pressure greater than minimum tolerated blood pressure (MTBP) (see Methods for further details on pressor titration). For patients who required phenylephrine, patients were divided into two groups by maximum dose required to maintain MTBP. Phenylephrine doses <200 mcg/min are generally considered standard in the management of hypotension while doses > 200 mcg/min are considered higher than usual practice [19]. Figure 2 depicts the percentage of patients responding by phenylephrine requirement.

**Figure 3:**
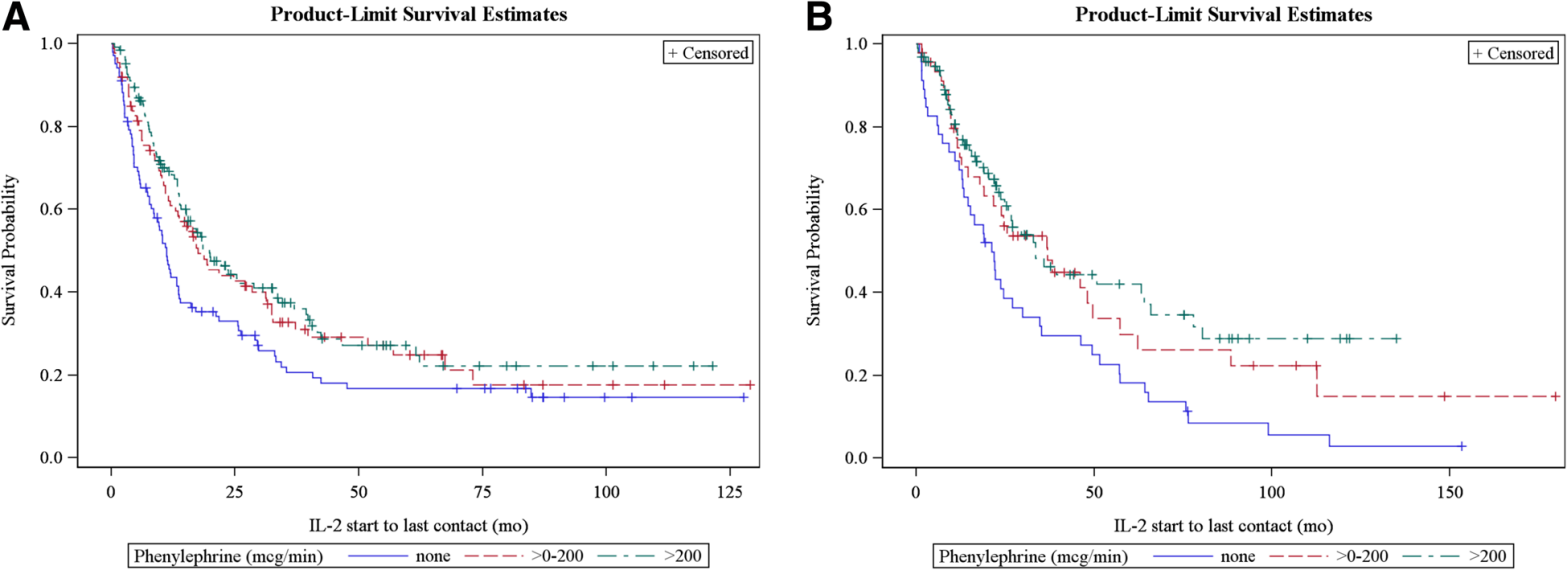
Survival by maximum phenylephrine pressor requirement (in mcg/min) in melanoma (A) and RCC (B).

In both melanoma and RCC, the proportion of patients with CR and PR increased significantly with increasing amounts of phenylephrine support of 0, >0-200, and >200 mcg/min (p = 0.003 and 0.0003, for the linear trend in response across phenylephrine dose groups for melanoma and RCC, respectively). Figure 3 shows survival by phenylephrine requirement and diagnosis. Survival was not diminished by requirement for pressor support, even at the highest levels, during IL-2. Since response occurred in a higher proportion of patients requiring phenylephrine, survival was also statistically significantly better in both melanoma (p = 0.018) and renal cancer (p = 0.007), compared to patients who required no pressor support. A similar analysis was done adjusting the phenylephrine dose by patient weight (mcg/kg/min) and there was no difference in the response or survival results as summarized above.

Metabolic acidosis defined by decreased serum bicarbonate levels is another severe IL-2-related toxicity that can arise from lactic acid production by proliferating T cells [20]. The acidosis is exacerbated by compromised homeostatic mechanisms from decreased hepatic and renal function during IL-2. Acidosis can also result from poor tissue perfusion during episodes of hypotension. Due to our management strategy of repleting bicarbonate when serum levels were less than 20 mmol, the majority of patients achieved normal serum bicarbonate levels within 12 hours after starting repletion. Table 3 shows response by bicarbonate nadir. Complete and partial response rates were significantly greater in patients with bicarbonate in the 15–19 mmol range in melanoma (p = 0.010) and in RCC (p = 0.001).

**Table 3:** Response in melanoma and RCC by bicarbonate nadir

	**Melanoma**			**Renal cell carcinoma**		
Bicarbonate (mmol)	<15	15-19	>19	<15	15-19	>19
Total N (%)	8 (100)	258 (100)	45 (100)	10 (100)	152 (100)	20 (100)
CR, PR	1 (13)	80 (31)	5 (11)	6 (60)	37 (24)	1 (5)
SD, PD	7 (88)	178 (69)	40 (89)	4 (40)	115 (76)	19 (95)

Others have reported that thrombocytopenia correlates with response to high-dose IL-2 [14,21]. We analyzed our results according to the platelet nadir during any treatment cycle. In both melanoma and RCC there was a statistically significant linear trend between achieving CR or PR and lower platelets counts of < 50,000 cells/mm3; 50,000-100,000; compared to > 100,000 (p = 0.024 and p = 0.022 for melanoma and RCC, respectively).

There were 5 deaths that occurred during IL-2 therapy in the hospital. Three of the deaths were in patients who were not hypotensive, while 2 patients who died were hypotensive during their IL-2 hospitalization and required phenylephrine at a dose >200 mcg/min; however, they were neither hypotensive nor on pressors when death occurred. Two deaths were attributable to severe IL-2 toxicities (pulmonary capillary leak (n = 1) and neurocortical toxicity (n = 1)). The other deaths were from progressive disease (n = 2) and an adverse event unrelated to IL-2 (air embolism from a patient who removed the central venous catheter without other mental status changes (n = 1)). No patient died from toxicity related to phenylephrine. Two patients experienced bowel perforation repaired surgically. Both patients survived the operation and were discharged from the hospital.

The maximum number of IL-2 treatment cycles is generally 6 for responding patients due to the earlier onset and severity of toxicities that necessitate holding IL-2 doses. Each cycle is defined as the 5-day hospital admission during which IL-2 is administered. Two cycles comprise 1 course of IL-2 (see Methods for further details). The number of doses administered to responding patients during the first 6 cycles is depicted in Table 4, which shows the general downward trend in the median number of IL-2 doses administered per treatment cycle.

**Table 4:** Responders’ median number of IL-2 doses by cycle and diagnosis*

**Cycle**	**Melanoma**		**Renal cancer**	
	**N**	**Median # doses**	**N**	**Median # doses**
1	86	12	44	11
2	86	10	44	8
3	84	10	43	9
4	84	7	43	5
5	58	8	23	6
6	55	4	18	4

*One responding patient with RCC received 8 cycles.

**Figure 4:**
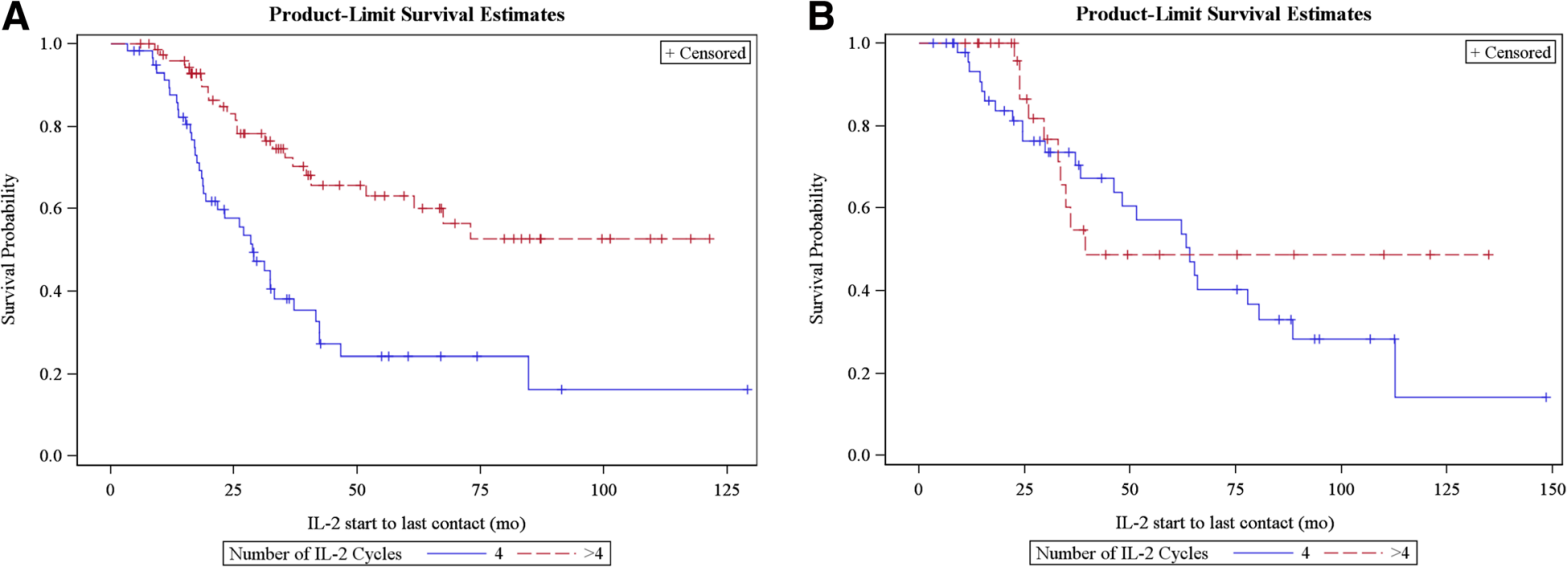
Survival by number of IL-2 cycles in melanoma (A) and RCC (B).

The mean number of IL-2 doses in the first 2 cycles in patients who had a best overall response of CR or PR versus SD or PD was similar (melanoma responder vs non-responder mean = 21.1 SD = 3.7 vs mean = 19.5 SD = 4.3 and RCC mean = 18.5, SD = 3.7 vs mean = 17.7 SD = 4.7). Although 6 IL-2 cycles is a practical maximum for patient tolerability, there was also variation in clinical practice among physicians and patient preferences for receiving cycles 5 and 6 if ongoing response was manifest after 4 cycles. Figure 4 shows overall survival by the maximum number of cycles administered in melanoma and RCC of the patients that received at least 4 cycles of IL-2. Survival rates were greater for patients with melanoma who received >4 versus 4 cycles (n = 58, 74; p < 0.0001), but there was no difference in RCC (n=49, 30; p = 0.39).

One of the observations in early clinical trials of IL-2 was that some partial as well as complete responses were durable without the administration of additional systemic therapy. We also wanted to characterize the survival of patients who received cancer treatment after IL-2. We had treatment follow-up data for 399 patients (252 with melanoma and 147 with RCC) after completion of IL-2 and survival data for all patients. No additional therapy was needed in 21% of patients with melanoma (53/252) and 22% in RCC (33/147). Table 5 depicts the best overall response by diagnosis for the patients who required no further medical therapy. Among these patients, only one death has been observed in a patient with RCC. For patients who went on to receive systemic medical therapy after IL-2, the median survival from start of IL-2 therapy was 18.4 months in patients with melanoma and 27.0 months in RCC. The median time to starting a new treatment after IL-2 was 3 and 5.1 months for melanoma and renal cancer, respectively. In patients with melanoma who received subsequent therapy, 44 were treated with ipilimumab and 6 with vemurafenib. The median survival of patients who enrolled in palliative care or hospice or who declined further medical therapy after IL-2 (due to disease progression) was 3.3 and 2.4 months for patients with melanoma and RCC, respectively.

**Table 5:** Best overall radiographic response for patients who did not require additional systemic therapy

	**CR**	**PR**	**SD**	**PD**	**Total**
Melanoma	27	17	7	2	53
Renal cancer	9	11	10	3	33

## Discussion

High-dose IL-2 has been available to treat patients with melanoma and renal cancer since the 1990’s. Despite the fact that long-term disease-free survival is seen in some patients, there are only approximately 100 cancer centers in the US that offer high-dose IL-2 because of concerns about toxicity, cost and doubts about efficacy. The skepticism about efficacy is a consequence of the original clinical development of IL-2 during which a randomized phase III study to prove there was a survival benefit compared to other treatments was never performed. The response rate and survival of patients with melanoma and RCC with high-dose IL-2 monotherapy reported here is comparable or superior to that described in other studies [14,22,23]. The patients with melanoma and RCC who had stable disease as their best response after IL-2 also had clinically significant survivals. Stable disease was not generally reported as an outcome in the 1980’s and 1990’s when the first clinical reports of IL-2 were published in the medical literature. It has been appreciated more recently that patients who have stable disease after immunotherapy can have clinically meaningful benefit from therapy. This has been illustrated extensively with ipilimumab in patients with melanoma [2,3,24]. The objective response among the patients who required no further therapy for their melanoma or RCC after IL-2 was predominantly CR or PR; however, some individuals had SD and a few PD. The individuals with PD on initial scans had minor radiographic abnormalities that at the time of evaluation were interpreted as cancer progression, but in retrospect were likely inflammatory changes. To our knowledge there are no long-term follow-up studies on IL-2 clinical outcomes published in peer-reviewed literature in the last decade. The 3-year survival of 31% we report in melanoma is greater than the 3-year survival reported after ipilimumab of 16% in one study [24]. A larger retrospective study reported a 5-year survival of 22% after ipilimumab, comparable to the 23% reported in our IL-2 patients [4]. Similarly, the 3-year survival in RCC of 44% is greater than that reported with VEGFTKI agents, for which the 3-year survival is 20 – 30% [12,25]. Although we describe a single institution experience, the total number of patients in this report is greater than other IL-2 single- or multi-institution studies in the medical literature. We believe these findings are significant in light of the recent strong interest in immunotherapy and the knowledge that the objective response rates for T-cell directed antibody monotherapy appear to be between 10 – 30%, which are comparable to our findings with IL-2.

We chose to examine the outcomes of our IL-2 patients in relation to hypotension, which is the main dose-limiting toxicity for this treatment. This perspective is the reverse of the paradigm used to assess most other medical treatments. Most oncologic agents are developed using phase I dose escalation studies with the primary objective of finding a tolerable and biologically active dose. The logic behind this drug development paradigm is that toxicity limits dosing, and limited dosing will decrease the efficacy of the agent due to decreased dose intensity. In addition, toxicity could also result in mortality or significant morbidity that would diminish long-term survival. For biologic agents that have a mechanism of action inseparable from the physiology of immune activation, this paradigm may not be valid. We show in our IL-2 population that there is improved response probability and survival in patients who experienced dose-limiting hypotension requiring high-dose phenylephrine pressor support. We are not implying a direct causal link between hypotension and tumor response, rather that treating patients to their individualized MTD with IL-2 results in durable remissions. In addition, our findings support that survival and objective response are not compromised by toxicity. There is one previous study with findings similar to ours showing a favorable correlation with response and IL-2-induced hypotension in melanoma patients [26]. The incidence of death reported here is comparable to other reports in the medical literature on IL-2 [13]. Others have noted that there may be an adverse interaction between prior treatment with VEGF-TKI therapy and interleukin-2 cardiac toxicity in patients with metastatic renal cancer [27]. There was no discernible difference in the severity or frequency of cardiac or other IL-2-related toxicities in the 6% of patients with RCC with previous VEGF-TKI described here. None of the deaths reported in this retrospective study occurred in patients with prior VEGF-TKI treatment.

At many immunotherapy centers, IL-2 is given in an intensive care unit setting, and multiple pressors are used to manage hypotension. Although the management of patients receiving IL-2 is complex, scheduling and administering cancer therapy in the ICU often strains scarce hospital resources, the patient’s care is transferred to a team that does not have extensive experience with cancer patients or specifically with IL-2. At our cancer center we administer IL-2 on the general oncology in-patient service, but have the ability to administer phenylephrine and place patients on continuous cardiac monitoring when needed. We manage IL-2 induced hypotension with phenylephrine because it is an alpha-adrenergic receptor agonist that has much less beta-adrenergic effect than dopamine or norepinephrine [28]. IL-2 can also cause tachydysrhythmias, so avoiding inotropes with the ability to increase heart rate is desirable. The general teaching in critical care medicine is that phenylephrine doses greater than 200 mcg/min (2 mcg/kg/min) do not confer significant additional inotropy; however, there is only one dose–response study in septic surgical patients in the recent medical literature [29]. We have acquired significant experience using phenylephrine at high doses (up to 5 mcg/kg/min) to manage IL-2-induced hypotension and it is well tolerated in this patient population preselected to have normal cardiopulmonary reserve at baseline.

The optimal number of IL-2 cycles to induce durable responses has not been defined in previous clinical trials. Our retrospective study suggests that responding patients with melanoma have enhanced long-term responses if they receive >4 IL-2 cycles; however, 4 cycles may be sufficient in patients with RCC. There are selection biases inherent in this retrospective analysis and determining the optimal number of IL-2 cycles could only be answered definitively in a randomized trial comparing 4 versus 6 cycles in responding patients with long-term follow-up. However, if there was no benefit of > 4 cycles in patients with melanoma, one might have expected the survival to be equivalent between these groups.

Although there are many newer agents that can be used to treat metastatic melanoma and renal cell carcinoma, it is our practice that IL-2 should be offered in the first or second line of therapy for patients who have normal baseline cardiopulmonary status with the goal of achieving durable regressions. In this retrospective analysis, there are 53 patients with melanoma and 33 with RCC who remain alive, free of disease and have not required additional systemic therapy. Although some of these patients will likely need systemic therapy in the future, at a minimum, the delay in the need for medical therapy will allow the development of more effective agents that can be offered when needed. Many of our patients who had disease progression after IL-2 as first line therapy participated in clinical trials for second or third line therapy. For melanoma, clinical trials using T-cell directed antibodies including ipilimumab and anti-PD-1 were offered. In renal cancer, treatment after IL-2 was most commonly a VEGF-TKI agent via clinical trial or standard of care. We believe these subsequent therapies had a favorable influence on the survival data presented here.

IL-2 can be administered to patients whose cancers have progressed after other agents. We believe this approach is not optimal especially in patients with renal cancer as durable remissions are rarely achieved with TKI therapy, and the patient’s performance status is more likely to decline with each successive systemic treatment, thus the opportunity to use IL-2 can be lost. The correspondence between good performance status (ECOG 0–1) and IL-2 response has also been observed by others [30,31]. This retrospective study confirms that IL-2 can be administered safely in the community setting, that severe toxicities can be managed with a well-trained biotherapy team and that excellent clinical results with durable responses can be achieved in melanoma and RCC. Our observations support that patients who receive high-dose IL-2 should be treated to their individualized MTD to derive the greatest clinical benefit from this immunotherapy.

## Conclusions

High-dose IL-2 can be administered safely, severe toxicity is reversible and does not compromise objective response rate. The tumor response and survival reported here after IL-2 are superior to the published literature and confirms that durable regressions of disease are achievable in patients with advanced melanoma and renal cancer. Our findings also support the practice of treating patients to their individualized maximum tolerated IL-2 dose. IL-2 should remain part of the treatment paradigm in selected patients with melanoma and RCC.

## Methods

### Selection of patients

All patients had a diagnosis of either metastatic RCC or metastatic melanoma and had signed informed consent for inclusion in the Providence Cancer Center Biotherapy Program database between 1997 and December 2012. All 1601 admissions during this time interval were examined. Patients who receive high-dose IL-2 must first have a pre-treatment evaluation including pulmonary function testing, laboratory tests of hepatic and renal function and cardiac stress testing when applicable, to assess their ability to withstand the toxicity of treatment. A brain MRI or other brain imaging is also included in the evaluation of patients with melanoma or RCC patients with symptoms suggestive of CNS metastases. Patients with treated brain metastases received IL-2 therapy after completing radiation and/or surgery, and were off corticosteroids for a minimum of 2 weeks. Patients with autoimmune disease requiring active therapy were excluded. Interleukin-2 Regimen: Patients were admitted to the medical oncology unit of Providence Portland Medical Center. The care team consists of a biotherapy attending physician, nurse practitioner and oncology certified staff nurses who have received specific didactic training and supervised preceptor experiences in the management of patients receiving high-dose IL-2 and in the titration of vasopressors. EKG telemetry, oximetry and continuous blood pressure monitoring is readily available for patients with hemodynamic instability. IL-2 (Prometheus Pharmaceuticals, San Diego, CA) was administered at 600,000 international units/kg/dose by IV bolus every 8 hours for a maximum of 14 doses followed by a 16-day rest period, followed by a repeat cycle. IL-2 doses were held for severe toxicity, but there was no reduction in the calculated amount per dose. Additional IL-2 was offered to patients having regression of disease. One patient received 8 cycles, but 6 cycles was maximum offered to responding patients. Additional courses (one course of therapy is defined as two cycles) of high-dose IL-2 were administered on average, within 9 weeks of completion of the prior course. Additional time off between courses of therapy was considered on a case-by-case basis to allow adequate recovery.

All antihypertensive medications were discontinued before hospital admission. A triple lumen central venous catheter was placed at the beginning of each cycle of IL-2 and removed before hospital discharge. Patients received antibiotic prophylaxis to reduce infection. Patients were routinely monitored and received supportive care for management of toxicities experienced as a consequence of therapy. We used our Biotherapy Program standard operating procedures for management of IL-2 toxicities, which are based on other published guidelines, but differ significantly in that high-dose phenylephrine is used when needed and IL-2 doses are rarely held for acute renal insufficiency or metabolic acidosis [32]. Before starting IL-2, a MTBP was defined, usually systolic 85–90 mmHg, based on a clinical judgment of the patient’s physiological reserve as indicated by ETT and baseline blood pressure. If the patient’s systolic BP fell below the MTBP, a normal saline fluid bolus was administered over 15 minutes. If the blood pressure did not rise to > MTBP, then the NS bolus was repeated up to two additional times. If the blood pressure remained below the MTBP after 3 NS boluses, treatment with phenylephrine was initiated. A small number of patients in the database also received dopamine pressor support in addition to phenylephrine, but are not reported separately.

### Titration of phenylephrine

The initial phenylephrine dose was 40 mcg/min with rapid titration in increments of 25–50 mcg every 5–15 minutes to achieve the MTBP. The minimum amount of phenylephrine was then used to maintain the MTBP. Patients who required more than 200 mcg/min sometimes received additional interventions such as fluid boluses. If the phenylephrine dose exceeded 5 mcg/kg/min to maintain the MTBP, the patient was transferred to the ICU, where additional vasopressors (e.g.: norepinephrine) or other interventions were implemented as dictated by the patient’s clinical needs. Doses of IL-2 were held during hypotensive episodes if the phenylephrine dose was ≥ 100 mcg and/or if the titration requirement for phenylephrine was increasing in the hour prior to the planned IL-2 dose. IL-2 was resumed if the dose of phenylephrine was <100 mcg/min, the titration trend was downward, and there were no other dose-limiting toxicities. IL-2 was not generally discontinued for phenylephrine doses peaking >200 mcg/min in contrast to other centers [32].

### Tumor response

Computed tomography was the most commonly used imaging modality to assess tumor response and was usually obtained after every 2 IL-2 cycles and every 3 months for the first year after IL-2 was completed. Imaging obtained after this interval was at the discretion of the attending physician and the clinical circumstances of the patient. Response evaluation criteria for solid tumors (RECIST) and the assessment of the attending physician were used to categorize response [33]. Complete response (CR) was defined as the complete disappearance of all target and non-target lesions. Partial response (PR) was defined as a 30% or greater decrease in the sum of the maximum diameter of target lesions. Progressive disease (PD) was defined as a 20% or greater increase in the sum of the largest diameter of target lesions or the appearance of new lesions. Stable disease (SD) was assigned to patients who did not meet criteria for the other response designations. Patients with minor regression (e.g.: tumor response not sufficient to qualify for PR) could receive additional IL-2 cycles if clinical benefit was present as assessed by the attending physician. Radiology reports were available for all patients and responses were reviewed by the biotherapy attending physician (BDC), but were not reviewed by an independent radiologist for this retrospective analysis.

### Statistical analysis

Analyses were carried out separately for melanoma and renal cancer patients due to marginal evidence of interaction effects between measures and cancer type for both best response and survival outcomes. Each interaction was tested in a separate model. For best response, logistic regression was used, and for survival, Cox proportional hazards was used. Best response was determined from assessment across all scans done after completing each IL-2 course, typically about 4 weeks later. Association between best response (CR+PR, SD+PD) and phenylephrine dose group (none, >0 – 200, >200 mcg/min; none, >0 – 2.5, >2.5 mcg/kg/min), platelet nadir group (<50, 50–100, >100 thousand), bicarbonate nadir group (<15, 15–19, >19 mmol/L), and IL-2 cycles [1-8] were tested with the Cochran- Armitage test for trend. Exact Cochran-Armitage test was used for bicarbonate nadir in the renal group due to small expected number of observations, and Fisher’s Exact in the melanoma group due to small expected number of observations and non-linear association with response. Effect of phenylephrine dose rate group was analyzed both with and without adjustment for patient weight. Time from start of first IL-2 dose to death was analyzed using survival analysis. Median duration of follow-up was modeled with deaths censored. Log rank tests were used to test for differences in Kaplan-Meier (product limit) survival estimates among phenylephrine dose groups, with and without adjustment for body weight, groups of number of IL-2 cycles, and whether subsequent treatment was received after IL-2. Analyses were performed using SAS 9.3 (SAS Institute Inc., Cary, NC).

## Abbreviations

BP: Blood pressure; CNS: Central nervous system; CR: Complete response; CT: Computed tomography; ECOG: Eastern cooperative oncology group; EKG: Electrocardiogram; ETT: Exercise tolerance test; FDA: Food and Drug Administration; ICU: Intensive care unit; IL-2: Interleukin-2; IU: International units; IV: Intravenous; Kg: Kilograms; LDH: Lactate dehydrogenase; L: Liter; MRI: Magnetic resonance imaging; MTD: Maximum tolerated dose; Mcg: Micrograms; Mg: Milligrams; mmHg: Millimeters mercury; mmol: Millimoles; m-TOR: Mammalian target of rapamycin; MTBP: Minimum target blood pressure; Min: Minute; NS: Normal saline; PR: Partial response; PD: Progressive disease; RCC: Renal cell carcinoma; RECIST: Response evaluation criteria in solid tumors; SD: Stable disease; TKI: Tyrosine kinase inhibitor; VEGF: Vascular endothelial growth factor.

## Competing interest

The study was supported by the Providence Cancer Center. There was no external research funding.

The authors have reported the following:

RP: No competing interests

HH: No competing interests

LG: No competing interests

BR: No competing interests

JS: No competing interests

RL: No competing interests

TC: No competing interests

WU: Paid consultant (BMS, Medimmune), Honoraria (BMS, Medimmune) Travel Expenses (BMS, Medimmune)

BC: Research funding (Prometheus Pharmaceuticals), Paid consultant (Prometheus Pharmaceuticals), Travel Expenses (Prometheus Pharmaceuticals, Agonox)

Institution: Research Funding (BMS, MedImmune, Prometheus Pharmaceuticals).

## Authors’ contributions

Conception and design: RP, LG, BC; Collection and assembly of data: RP, BR, LG, JS, RL, TC, WU, BC; Data analysis and interpretation: HH, BC; Manuscript writing and final approval of manuscript: RP, HH, LG, BR, JS, RL, TC, WU, BC. All authors approved and read the final manuscript.
